# Effect of Sexual Education on Sexual Function of Iranian Couples During Pregnancy: A Quasi Experimental Study

**Published:** 2018

**Authors:** Masumeh Heidari, Farkhondeh Aminshokravi, Farid Zayeri, Seyed Ali Azin

**Affiliations:** 1- Department of Health Education and Health Promotion, Faculty of Medical Sciences, Tarbiat Modares University, Tehran, Iran; 2- Faculty Member of Shahed University, Tehran, Iran; 3- Department of Biostatistics, Faculty of Paramedical Sciences, Shahid Beheshti University of Medical Sciences, Tehran, Iran; 4- Department of Health Promotion, Iranian Academic Center for Education, Culture and Research, ACECR, Tehran, Iran

**Keywords:** Couple, Pregnancy, Sex education, Sexual function, Third trimester

## Abstract

**Background:**

The aim of this study was to evaluate the impact of husbands' participation in sexual education on sexual function during pregnancy.

**Methods:**

This quasi experimental study was conducted on 123 couples who were divided in two intervention (A: couples, B: pregnant women) and one control (C) groups. Group A couples received sex education, Group B women received sex education without their spouses, and Group C women received routine prenatal care without sex education. Sexual functions of couples were assessed by Female Sexual Function Index (FSFI) and International Index Erectile Function (IIEF) questionnaires, before sex education, four weeks after the intervention, at the end of the second trimester and at the end of the third trimester.

**Results:**

Mean total scores of FSFI and IIEF were not different at baseline in three groups. Repeated measure analysis showed significant differences between groups (A and B with C) in the mean total scores of FSFI and IIEF during the third trimester. The mean total scores of the two intervention groups of A and B were not significant.

**Conclusion:**

According to the results of the present study, promoting the sexual function of pregnant women needs to include the sex education on prenatal care. Whereas spouses’ participation was suggested to have a great role in the effectiveness and strengthening of the education in various studies, this study showed that the lack of spouses’ participation for whatever reasons may lead to the same results of previous studies which emphasized the necessity of spouses’ participation.

## Introduction

Despite social and cultural influences, different life conditions such as physiological and anatomical changes during pregnancy could affect the spouses' sexual life (1–4). Sexual satisfaction of couples and their happiness on marital relationship during pregnancy is an important public health issue (5). The prevalence of sexual dysfunction during pregnancy has been reported to be 46.6% in the first trimester, 34.4% in the second trimester and 73.3% in the third trimester (6, 7). Also, the prevalence of male sexual dysfunction during the pregnancy of partner has been reported to be 21.3% in the first trimester, 19.3% in the second trimester and 28.3% in the third trimester in Iran (8). Male sexual dysfunction during their wives’ pregnancy included premature ejaculation, sleep ejaculation, masturbation, impaired erection, altered sex drive and inability to reach orgasm (9, 10). As a result of these problems, various studies reported occurrence of extramarital relationships of men during their wives’ pregnancy (11–13) as they do not consider their wives' emotional and physical needs. In the case of physiological pregnancy, there is no limitation on the couples sex activity, unless high risk pregnancy has been diagnosed (14, 15). The normal trend in the first trimester of pregnancy is the decrease in sexual desire of women which results in reduction of the number and duration of the coitus because of the fear of increasing the risk of abortion or infection. In the second trimester, because of the stability of women’s condition and the decrease in their fear, sexual interest usually increases. The third trimester is usually characterized by a decrease in women sexual activity (16–18). Intimacy and sex help to provide feelings of happiness, pleasure, closeness, and vitality. Considering the increase of intimacy needs of pregnant women, the spouses should pay more attention to their wife, but due to lack of sex education interventions for couples, the women usually receive no response and subsequently the result is rigidity which may lead into separation of couples (19). Couples are not provided with information about how they can manage their sexual life during pregnancy (2, 14, 20–24) since midwives and obstetricians routinely do not conduct sexual health education (25, 26). Also, the literature reported the results of the cross-sectional or retrospective rather than prospective study design and only a limited number of interventional studies have been conducted (27). In this paper, the results of the second phase of a longitudinal study was reported which evaluated the effects of sex education sessions on sexual function of pregnant women and husbands in the third trimester of pregnancy (28).

Due to lack of adequate information, the three grouping interventions were designed to assess the impact of the partners' participation in sexual education classes on couples’ sexual function. The purpose of the study was examining the impact of sex education with husbands' participation on sexual function of the couples in the third trimester of pregnancy.

## Methods

### Participants:

In this quasi experimental study, participants included 128 nulliparous pregnant women and their spouses were referred to a public health center in Tehran, Iran. The inclusion criteria were prim-gravid women being in the 10–12th week of pregnancy living permanently with a spouse and single pregnancy. The research was conducted over a 12-month period in 2016 in Tehran, Iran. Exclusion criteria were no history of medical diseases in the couples, no medication, no smoking and lack of coitus for any medical problem.

### Allocation:

The participants were assigned into three groups of A, B and C. Group A (n=40) who were the couples (pregnant women with their spouses) received routine prenatal care and sex education. Group B (n=42) were only pregnant women who received routine prenatal care and sex education and group C (n=41) included pregnant women who received only routine prenatal care and no sex education.

The intervention was performed by a trained midwife. After giving a written consent, each participant completed a coded unknown pre-test questionnaire in a suitable place. Then, their address and contact details were collected for the follow ups. The couples in two intervention groups and the control group completed the questionnaires of sexual function (Female Sexual Function Index & International Index Erectile Function) and they participated in the pretest (10–12 weeks), four weeks after education, at the end of the second trimester (26–28 weeks) and at the end of the third trimester (34–36 weeks).

### Intervention:

Intervention groups A and B received the sex education in two sessions once a week for two consecutive weeks in the health centers.

In the intervention group A, the couples were trained together in one private room. Group B consisted of only pregnant women received sex education and control group C received routine prenatal care and no sex education. The education contents were developed based on the results of relevant previous studies and needs assessments of sex education for pregnant women in Iran and other countries (13, 27), as well as the information available in the written literature and interviews with specialists of sexology. The educational sessions (two) consisted of applied lectures, power points (including figures and plots) and the genital models. Each session lasted 90 minutes with one week interval, and some extra time to answer the questions was allocated.

In the first session, the topics including genital anatomy and sexual physiology consisting of orientation with erotic organs, sexual responses cycle, and the impact of pregnancy on sexual response cycle were discussed. In the second session, the impact of pregnancy on sexual behavior, sexual intercourse techniques, safe position during pregnancy, sexual skills, and common concerns related to sex such as the risk of miscarriage and PROM as a result of coitus during pregnancy were taught.

At the end of the first session, the written educational booklet was handed out to the pregnant women (in two groups A and B), then they were asked to study the contents together with their spouses and ask their probable questions in the next session and the educator contact number was given to them in order to answer their questions. Also, telegram which all pregnant women had access to was applied to send the contents about the sexual activities in pregnancy to couples. Learning via telegram by mobile continued during pregnancy until the end of the third trimester and in the meantime, the couples’ questions were answered.

Four weeks after the last educational session, at the end of the second-trimester (26–28 weeks of pregnancy) and at the end of the third-trimester (34–36 weeks of pregnancy), all participants were contacted and asked to complete the post-test questionnaire at the health care centers.

### Questionnaires:

Data collection tool consisted of questions about demographic data such as the participant’s age, educational level, job and income. Other questionnaires included the Female Sexual Function Index (FSFI), and International Index of Erectile Function (IIEF).

The FSFI is a valid and reliable questionnaire for evaluating the sexual function of women during the past four weeks. This questionnaire consists of 19 questions covering six different domains of sexual function, desire, arousal, lubrication, orgasm, satisfaction and pain. The score of each domain is calculated through adding the score of the individual items that comprise the domain, and multiplying the sum by the domain factor (sexual desire 0.6, sexual arousal and lubrication 0.3, orgasm, satisfaction and pain 0.4). The sexual desire score ranges from 1.2 to 6, and the rest of the domains score is determined by the sum of the six domains, and can vary from 2 to 36. Higher scores show better sexual function. The reliability of the FSFI has been approved by Mohammadi et al. (29) in Tehran, Iran, and Rosen et al. in other countries (30).

The International Index of Erectile Function (IIEF) is a multidimensional scale for assessment of erectile dysfunction (31). A structured interview, a standardized and validated 15-item selfevaluation scale provides evaluations of erectile function, orgasmic function, sexual desire, satisfaction in sexual intercourse and general satisfaction (32). Questionnaire reliability was evaluated through test re-test, which is done in two stages, with a two week interval. The Pearson’s correlation coefficient for functional domains was more than 0.85.

### Data analysis:

The sample size was calculated by using the PS (Power & Sample size calculation, version 3.1.2, 2014) (33) regarding M_1_=22.6, M_2_= 26.6, SD_1_=7.9 and SD_2_=8.4 based on the total score of the sexual function index from a study conducted in Tehran, Iran, (27) with type I error rate of 0.05 and statistical power of 90%. Assuming 10% loss to follow-up, 46 couples were assigned to each study group.

The quantitative and qualitative variables were described as mean (standard deviation) and frequency (percent), respectively. The normal distribution of data was checked through the Kolmogorov Smirnov’ test. ANOVA was used to compare mean differences among the three groups if the distribution was normal. Chi-square test was used to assess the relationship between qualitative variables. The repeated measures ANOVA was used to compare mean FSFI total scores, six domains and IIEF total scores during the study period in the three groups. All analyses were done using the SPSS^19.0^ (SPSS IBM, New York, USA), and P-values less than 0.05 were considered statistically significant.

### Ethical consideration:

This study was approved by the Ethics Committee of Tarbiat Modares University (IR.TMU.REC.2015.39). A written consent letter was obtained from all participants. They were told they could leave the research whenever they wanted and it does not affect their routine care. This trial is registered on www.Irct.IR (IRCT2016101930388N1).

## Results

**Figure 1.**
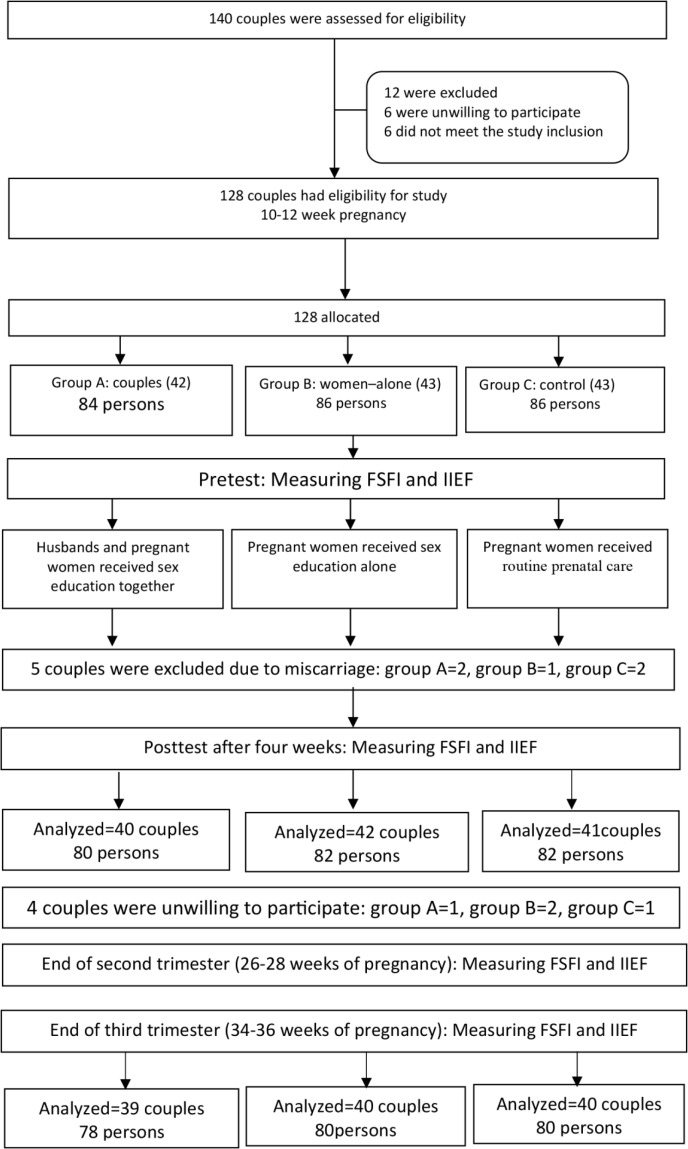
Flow diagram of the participants’ selection process

A total of 128 eligible pregnant women and their husbands were included in the study. Participants were allocated to one of the three groups of intervention A including 42 couples, intervention B including 43 couples, and control (C) including 43 couples. Five couples were excluded from the study due to miscarriage (End of the first trimester), two from the intervention group A, one from the intervention group B, and two from the control group. Also, four couples were excluded from the study due to unwillingness to participate (End of the second trimester), one from the intervention group A, two from the intervention group B, and one from the control group (Figure 1).

No significant differences were found in demographic variables of the subjects (28). The one way ANOVA showed that there were no significant differences among three groups in terms of FSFI and IIEF mean scores before training (Table 1 and 2).

**Table 1.** Trend of FSFI total scores and six domains in different groups (A=40, B=42, C=41)

**Domains of FSFI**	**Groups**	**First trimester (10–12 weeks)**	**4 weeks after intervention**	**Second trimester (26–28 weeks)**	**Third trimester (34–36 weeks)**	p ^**^
**Desire**						
	Group A	3.07 (1.17) ^*^	3.83 (0.88)	3.78 (1.09)	3.15 (0.97)	Time 0.001
	Group B	3.45 (1.23)	3.82 (0.79)	3.66 (0.82)	3.33 (0.97)	Time-Group 0.009
	Group C	3.00 (1.21)	2.95 (1.04)	3.55 (1.21)	3.09 (1.29)	Group 0.063
	p ^***^	0.193	0.001	0.629	0.597	
**Arousal**						
	Group A	3.86 (1.79)	4.02 (0.97)	4.06 (1.14)	3.33 (1.27)	Time 0.001
	Group B	3.22 (1.70)	4.15 (1.05)	4.07 (1.11)	3.33 (0.97)	Time-Group 0.009
	Group C	2.63 (1.82)	2.75 (1.65)	3.64 (1.47)	2.67 (1.61)	Group 0.003
	p ^***^	0.312	0.001	0.221	0.38	
**Lubrication**						
	Group A	3.76 (2.03)	5.067 (0.95)	4.90 (1.05)	4.05 (1.64)	Time 0.001
	Group B	4.04 (1.91)	5.20 (0.80)	4.83 (1.18)	4.23 (1.48)	Time-Group 0.045
	Group C	3.86 (1.81)	3.46 (1.85)	4.29 (1.54)	3.93 (2.79)	Group 0.016
	p ^***^	0.798	0.001	0.69	0.808	
**Orgasm**						
	Group A	3.55 (1.96)	5.11 (1.17)	4.44 (1.26)	3.47 (1.53)	Time 0.001
	Group B	3.74 (1.88)	4.65 (1.05)	4.58 (1.28)	3.96 (1.51)	Time-Group 0.001
	Group C	2.84 (1.82)	2.83 (1.81)	3.80 (1.59)	3.31 (1.58)	Group 0.001
	p ^***^	0.079	0.001	0.031	0.140	
**Satisfaction**						
	Group A	4.05 (1.56)	4.98 (0.87)	4.58 (1.14)	3.94 (1.47)	Time 0.001
	Group B	4.15 (1.58)	5.00 (0.96)	4.87 (0.96)	4.25 (1.29)	Time-Group 0.001
	Group C	3.88 (1.59)	3.55 (1.61)	4.54 (1.18)	4.04 (1.26)	Group 0.016
	p ^***^	0.736	0.001	0.327	0.595	
**Pain**						
	Group A	3.79 (2.19)	4.05 (0.97)	4.70 (1.24)	3.80 (1.63)	Time 0.001
	Group B	4.07 (1.83)	4.94 (0.90)	4.80 (1.15)	4.06 (1.57)	Time-Group 0.032
	Group C	3.65 (1.88)	3.34 (1.96)	4.59 (1.36)	3.63 (1.76)	Group 0.039
	p ^***^	0.617	0.001	0.75	0.51	
**Total**						
	Group A	22.35 (9.90)	27.32 (4.02)	26.70 (5.31)	21.77 (7.60)	Time 0.001
	Group B	22.70 (9.04)	27.78 (3.91)	26.84 (5.32)	23.49 (6.96)	Time-Group 0.001
	Group C	20.23 (8.58)	19.30 (8.39)	24.02 (7.23)	21.17 (8.71)	Group 0.002
	p ^***^	0.423	0.001	0.123	0.552	

Possible score for all domains were 0–6 except for desire which was 1.2–6.0 and for the total function was range 2–36.

* M (SD),

** From repeated measure test,

*** From ANOVA test

**Table 2.** Trend of IIEF total scores and five domains in different groups (A=40, B=42, C=41)

**Domains of IIEF**	**Groups**	**First trimester (10–12 weeks)**	**4 weeks after intervention**	**Second trimester (26–28 weeks)**	**Third trimester (34–36 weeks)**	p ^**^
**Desire**						
	Group A	7.33 (1.87) ^*^	7.55 (1.53)	7.46 (1.77)	6.58 (1.85)	Time 0.366
	Group B	7.07 (1.79)	7.64 (1.57)	7.67 (1.45)	7.17 (1.72)	Time-Group 0.009
	Group C	7.56 (1.8)	7.63 (1.57)	7.60 (1.46)	6.72 (1.88)	Group 0.049
	p ^***^	0.485	0.957	0.829	0.329	
**Erection**						
	Group A	21.7 (6.88)	26 (3.11)	26.12 (3.59)	3.33 (1.27)	Time 0.001
	Group B	22.16 (7.11)	25.61 (3.71)	25 (4.36)	3.33 (0.97)	Time-Group 0.181
	Group C	22.36 (8.22)	23.09 (7.63)	25.22 (4.64)	2.67 (1.61)	Group 0.529
	p ^***^	0.918	0.027	0.457	0.586	
**Orgasm**						
	Group A	7.27 (2.86)	8.35 (1.23)	8.43 (1.72)	7.02 (2.38)	Time 0.001
	Group B	7.45 (2.68)	8.64 (1.26)	8.52 (1.17)	7.72 (1.94)	Time-Group 0.045
	Group C	7.09 (3.06)	7.29 (2.70)	8.12 (1.47)	6.92 (2.29)	Group 0.016
	p ***	0.854	0.003	0.447	0.218	
**Satisfaction of coitus**						
	Group A	9.60 (4.22)	11.25 (2.15)	11.30 (2.27)	8.84 (3.53)	Time 0.001
	Group B	10.14 (3.57)	11.50 (1.96)	11.35 (1.90)	10.72 (2.83)	Time-Group 0.562
	Group C	9.04 (4.64)	9.53 (4.3)	10.52 (30.07)	8.15 (3.64)	Group 0.529
	p^***^	0.491	0.007	0.247	0.049	
**Total satisfaction**						
	Group A	7.62 (2.22)	8.45 (1.6)	8.35 (1.75)	7.33 (2.14)	Time 0.001
	Group B	8.21 (2)	8.76 (1.33)	8.50 (1.37)	7.75 (1.76)	Time-Group 0.734
	Group C	7.56 (1.87)	8.24 (1.94)	8.27 (1.41)	7.60 (1.77)	Group 0.274
	p ^***^	0.277	0.356	0.08	0.613	
**Total**						
	Group A	56.72 (8.04)	61.85 (6.25)	62.56 (6.47)	54.46 (9.59)	Time 0.001
	Group B	55.64 (11.11)	61.45 (7.83)	61.38 (7.71)	55.68 (12.31)	Time-Group 0.177
	Group C	55.10 (16.29)	54.83 (16.27)	56.68 (11.63)	52.78 (11.37)	Group 0.049
	p ^***^	0.833	0.007	0.009	0.508	

* M (SD),

** From repeated measure test,

*** From ANOVA test

**Figure 2.**
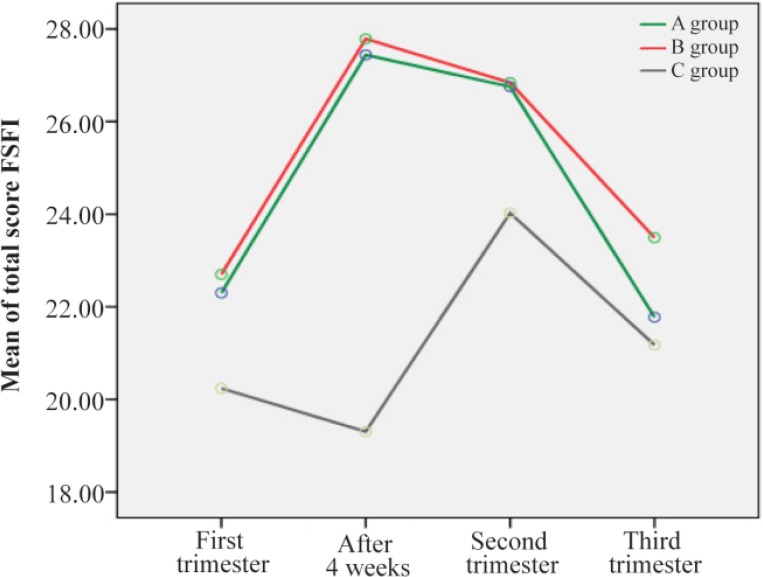
Trend of mean FSFI scores during the study period in three groups

Table 2 shows the descriptive statistics and results of repeated measures ANOVA for comparing mean FSFI total scores and six domains during the study period in the three groups. The effects of time (p<0.001), group (p<0.01) and interaction between time and group (p=0.001) were statistically significant. These results show significant differences between groups in mean FSFI total scores during the study period. As displayed in figure 2, the highest difference could be observed in the time of 4 weeks after intervention (a mean difference of eight scores between intervention groups (A and B) with the control group), while the minimum mean difference was related to the third trimester (non-remarkable difference between groups A and B with the control can be seen in this time point).

In six domains of FSFI during the study period in the three groups, the p-values in the last column of table 2, show that the effects of time, group and interaction between time and group (p<0.05) were statistically significant (Table 1).

**Figure 3.**
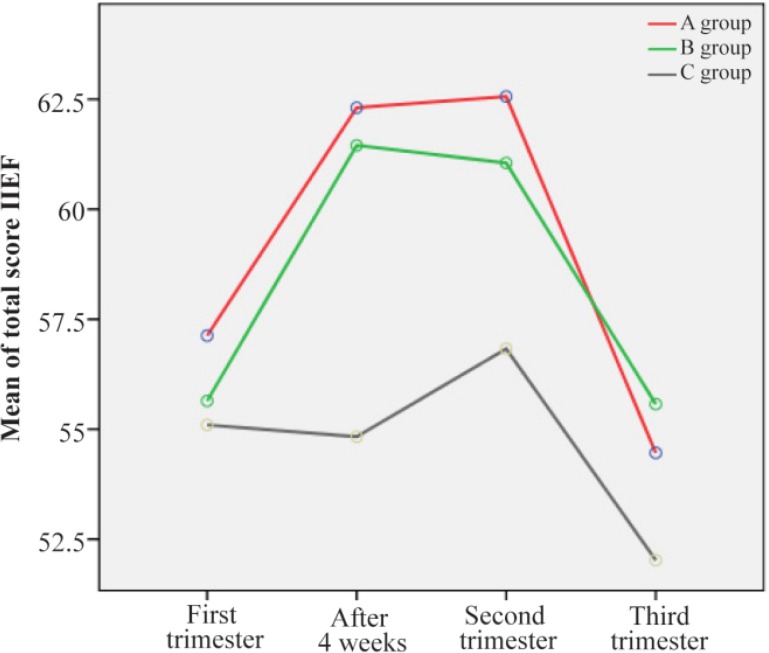
Trend of mean IIEF scores during the study period in three groups

Table 2 shows the descriptive statistics and results of repeated measures ANOVA for comparing mean IIEF total scores and five domains during the study period in the three groups. Also, the p-values in the last column of table 2 show that the effects of time (p=0.001), group (p=0.177) and interaction between time and group (p=0.049) were statistically significant. These results show significant differences between groups in mean IIEF total scores during the study period. As displayed in figure 3, the highest difference could be observed in the time period of 4 weeks after intervention (a mean difference of seven scores between intervention groups (A and B) with the control group), while the minimum mean difference was related to the third trimester (non-remarkable difference between group A and B with control can be seen in this time point).

## Discussion

The results of this study determine an improvement in the sexual function of couples due to the education offered. Couples' sexual function demonstrated positive changes in two intervention groups compared with the control group in four weeks after education and the second trimester only for husbands but in the third trimester, three groups were almost similar. This finding has been supported by some previous researches, which indicated improvements after sexual education (22–23, 27–28, 34–35). However, Wannakosit et al. showed no statistically significant differences between the two intervention groups and the control group after education (36). This disagreement of our study with the results of this study could be due to different demographic characteristics of the studied population, educational tools and methods used for the education. Longer duration of the present study could be one of the most important differences of the two mentioned studies (the number of sessions in our study was two sessions, each 90 *min*, compared to long sessions of 20 *min* in the cited study). Face to face learning as an individualized learning approach might help the couples to express their sensitive problems more easily and, consequently, receive more careful answers. This point is documented in the study of Moradi et al. who reported that education could not have any improvement on the sexual functions of the couples due to cultural limitations, group education in educational classes, as well as limitation in clear expression of sexual problems, which were well considered and removed in the present study (37)*.*

Mean differences in terms of the six domains scores of FSFI in the three groups between pretest and posttest were significant. The significant improvements seen in this study in some of the subscales of sexual female function are also supported (27). Although the mean scores of the six domains of FSFI in the second and the third trimester were not significant in three groups, in the two intervention groups, they were higher than the control group. It is reported in the other studies and some of text books (11, 38–40) that sexual function normally decreases during pregnancy, especially in the first trimester, due to fatigue, nausea, vomiting, emotional changes, and fear of abortion. During the second trimester, women have more energy, sexual desire, and vaginal lubrication that decrease the physical discomfort. It is probable that, during this period, pregnant women may reach orgasm for the first time or find it easier than before the pregnancy (39). During the third trimester of pregnancy, because of occurrence of the problems like dyspnea, weight gain, and back pain, sexual activities are more undesirable (10, 41). In the present study, the pattern of the sexual trend in the control group was the same as the trend mentioned above (Figure 2) and surprisingly was the same for the spouses (Figure 3). In the two intervention groups of the present study, despite the first trimester problems discussed above as a result of the education which resulted in the increase in sexual information and improvement in the sexual skills and fear reduction, sexual function of the couples increased significantly. In the second trimester, it was flat but in the third trimester as a result of fear of premature rupture of membrane, premature labor and fear of fetus damage, the sexual function, despite the continuous electronic learning decreased again and unsophisticated education was also one of the causes in this reduction. As it is reported, this kind of unscientific advice may create coldness and distance among couples (26). In the present study, the spouses stated that for the fear of fetus injery, they tried to have less intercourse, which is supported by other studies (8, 9, 42). Sexual activity is a mutual relationship and a change in women’s sexual function can affect the performance of men’s sexual needs and may result in an increase in sexual disorders among the couples (9). Many previous studies have suggested that sexual education during pregnancy with the spouses’ participation can improve the sexual function of the couples (2, 14, 23). Therefore, it seems that sexologist and midwife training should be synchronized and team working should be practiced for providing appropriate prenatal care for couples. De Pierrepont emphasizes that inter-disciplinary health care teams are future models of health care. In this model, the sexologist has a unique and important role, particularly in perinatal health care where sexuality is a central component of health and sexo-perinatal interventions should be a part of holistic perinatal health care to improve an intimate relationship to have an ideal sexual life (25, 43). The results of the present study showed that in pregnant women who participated with their spouses in sex education classes and received educational booklet compared with the women who participated in sex education classes alone but studied the booklet with their partners, sexual function increased after the intervention compared to the control group. But the differences of group A and B were not statistically significant.

Individual face to face education, having privacy and the use of three study groups facilitate the procedure for measuring the effect of spouses’ participation. Through following the participations till the end of the pregnancy, it was possible to see the results of the intervention on the couple’s sexual function. Another point to mention is that sexual education in our country is a taboo. Therefore, it would be expected to have a high number of cases who reject to participate. However, in this study, only four couples did not continue their participation because of their unwillingness. Self-report method of completing the questionnaire could cause over- or underestimation of the results and limit the study findings.

## Conclusion

It is recommended that sex education be integrated into prenatal care. In this study, the only difference between two intervention groups was the presence of spouses in the education class or not. And as such, regarding spouses’ participation in contrast with no participation of them, the result showed that sexual function of the couples in both groups was improved in the same way and there was no significant difference in sexual function of couples in the two intervention groups (A and B). As a result, it is possible to educate only pregnant women and hand out the educational material to them, to bring home and study with their spouses (indirect education of the spouses) (of course in a situation of good intimacy relationship). Then indirect education could lead to some benefits of less time and resources allocation and saving the national capitals.

## References

[1] Bartellas E Crane JM Daley M Bennett KA Hutchens D Sexuality and sexual activity in pregnancy. BJOG. 2000; 107( 8): 964– 8. 10955426 10.1111/j.1471-0528.2000.tb10397.x

[2] Gałązka I Drosdzol-Cop A Naworska B Czajkowska M Skrzypulec-Plinta V Changes in the sexual function during pregnancy. J Sex Med. 2015; 12( 2): 445– 54. 25378082 10.1111/jsm.12747

[3] Pauleta JR Pereira NM Graça LM Sexuality during pregnancy. J Sex Med. 2010; 7( 1pt1): 136– 42. 19845548 10.1111/j.1743-6109.2009.01538.x

[4] Pauls RN Occhino JA Dryfhout VL Effects of pregnancy on female sexual function and body image: a prospective study. J Sex Med. 2008; 5( 8): 1915– 22. 18547388 10.1111/j.1743-6109.2008.00884.x

[5] Nourani Sh Jonaidy E Shakeri MT Mokhber N Sexual satisfaction in fertile and infertile women attending state clinics in Mashad. J Reprod Infertil. 2010; 10( 4): 269– 77.

[6] Leite APL Campos AA Dias AR Amed AM De Souza E Camano L Prevalence of sexual dysfunction during pregnancy. Rev Assoc Med Bras. 2009; 55( 5): 563– 8. 19918657 10.1590/s0104-42302009000500020

[7] Jamali S Mosalanejad L Sexual dysfnction in Iranian pregnant women. Iran J Reprod Med. 2013; 11( 6): 479– 86. 24639782 PMC3941320

[8] Bayrami R Sattarzadeh N Koochaksariie FR Pezeshki MZ Sexual dysfunction in couples and its related factors during pregnancy. J Reprod Infertil. 2008; 9( 3): 271– 82.

[9] Ebrahimian A Heydari M Saberi Zafar Ghandi MB Delavari S Comparing Sexual dysfunctions in men before and during their wives' pregnancy. Iran J Obstet Gynecol Infertil. 2012; 15( 33): 19– 25.

[10] Nakić Radoš S Soljačić Vraneš H Šunjić M Sexuality during pregnancy: what is important for sexual satisfaction in expectant fathers? J Sex Marital Ther. 2015; 41( 3): 282– 93. 24512100 10.1080/0092623X.2014.889054

[11] Masters WH Johnson VE Human sexual response. 1st ed. Boston: Little, Brown and company; 1976 366 p.

[12] Nichols FH Humenick SS Childbirth education: practice, research and theory. 1st ed. US: Saunders Company; 2000 731 p.

[13] Onah HE Iloabachie GC Obi SN Ezugwu FO Eze JN Nigerian male sexual activity during pregnancy. Int J Gynecol Obstet. 2002; 76( 2): 219– 23. 10.1016/s0020-7292(01)00579-3 11818127

[14] Serati M Salvatore S Siesto G Cattoni E Zanirato M Khullar V Female sexual function during pregnancy and after childbirth. J Sex Med. 2010; 7( 8): 2782– 90. 20626601 10.1111/j.1743-6109.2010.01893.x

[15] Regan PC Lyle JL Otto AL Joshi A Pregnancy and changes in female sexual desire: A review. Soc Behav Pers. 2003; 31( 6): 603– 11.

[16] Aslan G Aslan D Kızılyar A Ispahi C Esen A A prospective analysis of sexual functions during pregnancy. Int J Impot Res. 2005; 17( 2): 154– 7. 15538394 10.1038/sj.ijir.3901288

[17] Malarewicz A Szymkiewicz J Rogala J [Sexuality of pregnant women]. Ginekol Pol. 2006; 77( 9): 733– 9. Polish. 17219804

[18] Jawed-Wessel S Herbenick D Schick V Fortenberry JD Cattelona GA Reece M Development and validation of the maternal and partner sex during pregnancy scales. J Sex Marital Ther. 2016; 42( 8): 681– 701. 26684371 10.1080/0092623X.2015.1113587

[19] Hassan ZR Shafie K Bashardoust N Reihani M Jaberi P Study of the related factors in couples sexual relationship during pregnancy. J Qazvin Univ Med Sci. 2002; 5( 4): 62– 7.

[20] Yeniel AO Petri E Pregnancy, childbirth, and sexual function: perceptions and facts. Int Urogynecol J. 2014; 25( 1): 5– 14. 23812577 10.1007/s00192-013-2118-7

[21] Read J Sexual problems associated with infertility, pregnancy, and ageing. BMJ. 2004; 329( 7465): 559– 61. 15345632 10.1136/bmj.329.7465.559 PMC516113

[22] Sossah L Sexual behavior during pregnancy: a descriptive correlational study among pregnant women. Eur J Med Res. 2014; 2( 1): 16– 27.

[23] Babazadeh R Mirzaii KM Masomi Z Changes in sexual desire and activity during pregnancy among women in Shahroud, Iran. Int J Gynecol Obstet. 2013; 120( 1): 82– 4. 10.1016/j.ijgo.2012.07.021 23073227

[24] Fok WY Chan LYS Yuen PM Sexual behavior and activity in Chinese pregnant women. Acta Obstet Gynecol Scand. 2005; 84( 10): 934– 8. 16167907 10.1111/j.0001-6349.2005.00743.x

[25] de Pierrepont C Polomeno V [Role of the perinatal sexologist in the interdisciplinary perinatal health care team in Canada]. Gynecol Obstet Fertil. 2014; 42( 7–8): 507– 14. French. 24961170 10.1016/j.gyobfe.2014.05.012

[26] Senkumwong N Chaovisitsaree S Rugpao S Chandrawongse W Yanunto S The changes of sexuality in Thai women during pregnancy. J Med Assoc Thai. 2006; 89 Suppl 4: S124– 9. 17725148

[27] Afshar M Mohammad-Alizadeh-Charandabi S Merghti-Khoei ES Yavarikia P The effect of sex education on the sexual function of women in the first half of pregnancy: a randomized controlled trial. J Caring Sci. 2012; 1( 4): 173– 81. 25276693 10.5681/jcs.2012.025 PMC4161090

[28] Heidari M Amin Shokravi F Zayeri F Azin SA Merghati-Khoei E Sexual life during pregnancy: effect of an educational intervention on the sexuality of Iranian couples: a quasiexperimental study. J Sex Marital Ther. 2017: 1– 11. [Epub ahead of print]. 10.1080/0092623X.2017.1313799 28368742

[29] Mohammadi Kh Heydari M Faghihzadeh S The female sexual function index (FSFI): validation of the Iranian version. Payesh. 2008; 7( 3): 269– 78.

[30] Rosen C Brown C Heiman J Leiblum S Meston C Shabsigh R The Female Sexual Function Index (FSFI): a multidimensional self-report instrument for the assessment of female sexual function. J Sex Marital Ther. 2000; 26( 2): 191– 208. 10782451 10.1080/009262300278597

[31] Rosen RC Riley A Wagner G Osterloh IH Kirkpatrick J Mishra A The international index of erectile function (IIEF): a multidimensional scale for assessment of erectile dysfunction. Urology. 1997; 49( 6): 822– 30. 9187685 10.1016/s0090-4295(97)00238-0

[32] Pakpour AH Zeidi IM Yekaninejad MS Burri A Validation of a translated and culturally adapted Iranian version of the international index of erectile function. J Sex Marital Ther. 2014; 40( 6): 541– 51. 24308814 10.1080/0092623X.2013.788110

[33] Dupont WD Plummer WD Jr Power and sample size calculations for studies involving linear regression. Control Clin Trials. 1998; 19( 6): 589– 601. 9875838 10.1016/s0197-2456(98)00037-3

[34] Mangeli M Ramezani T Mangeli S The effect of educating about common changes in pregnancy priod and the way to cope with them on marital satisfaction of pregnant women. Iran J Med Educ. 2009; 8( 2): 305– 13.

[35] Bahadoran P MohammadiMahdiabadzade M Nasiri H GholamiDehaghi A The effect of face-to-face or group education during pregnancy on sexual function of couples in Isfahan. Iran J Nurs Midwifery Res. 2015; 20( 5): 582– 7. 26457096 10.4103/1735-9066.164512 PMC4598905

[36] Wannakosit S Phupong V Sexual behavior in pregnancy: comparing between sexual education group and nonsexual education group. J Sex Med. 2010; 7( 10): 3434– 8. 20214714 10.1111/j.1743-6109.2010.01715.x

[37] Moradi Z Shafiabadi A Sodani M Effective communication training on marital satisfaction in mothers of elementary school students in the city of Khorramabad. Educ Res. 2008; 5: 114– 97.

[38] Polomeno V Sex and pregnancy: A perinatal educator's guide. J Perinat Educ. 2000; 9( 4): 15– 27. 10.1624/105812400X87879 PMC1595041 17273227

[39] Murtagh J Female sexual function, dysfunction, and pregnancy: implications for practice. J Midwifery Womens Health. 2010; 55( 5): 438– 46. 20732665 10.1016/j.jmwh.2009.12.006

[40] Corbacioglu A Bakir VL Akbayir O Cilesiz Goksedef BP Akca A The role of pregnancy awareness on female sexual function in early gestation. J Sex Med. 2012; 9( 7): 1897– 903. 22524554 10.1111/j.1743-6109.2012.02740.x

[41] Navidian A Rigi SN Soltani P Effects of group sexual counseling on the traditional perceptions and attitudes of Iranian pregnant women. Int J Womens Health. 2016; 8: 203– 11. 27366105 10.2147/IJWH.S104887 PMC4913995

[42] Liu HL Hsu P Chen KH Sexual activity during pregnancy in Taiwan: a qualitative study. Sex Med. 2013; 1( 2): 54– 61. 25356288 10.1002/sm2.13 PMC4184498

[43] de Pierrepont C Polomeno V Bouchard L Reissing E [What do we know about perinatal sexuality? a scoping review on sexoperinatality-part 1]. J Gynecol Obstet Biol Reprod (Paris). 2016; 45( 8): 796– 808. French. 27388468 10.1016/j.jgyn.2016.06.003

